# Hospital Meetings, &C.

**Published:** 1898-05-07

**Authors:** 


### HOSPITAL MEETINGS, &C.

### HAMPSTEAD HOSPITAL.

The festival dinner of the Hampstead Hospital, which is held every two years, took place at the Grand Hotel, Northumberland Avenue, on Wednesday, the 27th ult., Mr. T. SANSOME PRESTON presiding. The guests included Sir Henry Burdett, K.C.B., Messrs. E. Brodie Hoare, M.P., E. Bond, M.P., J. S. Fletcher, W. F. Malcolm (treasurer), E. K. Blyth, and several members of the medical staff.

After the loyal toasts and the "Houses of Parliament" (the latter proposed by Mr. J. S. FLETCHER, and responded to by Mr. E. BRODIE HOARE, M.P.) had been duly honoured,

The CHAIRMAN gave "Prosperity to the Hampstead Hospital and Nursing Institute." He said that he would not trouble them with statistics as to the work done by the hospital, but would content himself with saying that the number of out-patients treated in 1897 was larger than in any year since the hospital was founded, and was about two-fifths more than it was five or six years ago. After referring to the fact that the hospital now required rebuilding, he said that for many years after it was started it was called the Hampstead Home Hospital, and he could not help thinking that the use of that word "home" was a great comfort and solace to the patients. He hoped, therefore, that those who had the carrying out of the rebuilding scheme would bear that in mind, and would see that the new hospital was not a big, massive building, but rather that it was a homelike structure. (Applause.)

Mr. W. F. MALCOLM, in responding, hoped they would soon be able to rebuild the hospital on a new site, where would be convenient, both for the people of the older portions of Hampstead and for those of West Hampstead. The probable cost would be £10,000, and he expressed the hope that they might find a gentleman who would be willing to give this amount. (Applause.)

The HON. SECRETARY (Mr. R. A. Owthwaite) read out the list of contributions to the dinner fund, which amounted to £1,010, and said that he had received a letter from the last speaker (Mr. Malcolm), in which he promised to give £1,000 towards the rebuilding fund when the other £9,000 had been raised. Should, however, any gentleman give the whole £10,000, Mr. Malcolm would give £1,000 towards the equipment and endowment of the new hospital. (Applause.)

Sir HENRY BURDETT, K.C.B., proposed "The Health of the Council, Treasurer, and Hon. Secretary," and said that the Hampstead Hospital occupied almost a unique position amongst the hospitals of the metropolis, for the reason that it provided for the necessities and needs of the sick of a suburb of London, and that it owed its support and being to the inhabitants of that suburb. He had followed the fortunes of the Hampstead Hospital with a great deal of interest, as it was in a manner a sort of offspring of the Home Hospitals movement which he had started. Its career had demonstrated very clearly to him that at the present time public opinion in London was not sufficiently advanced to support what were known as middle-class hospitals—that was to say, hospitals for the class of patient who could pay less than a self-supporting rate for their treatment. He thought it well that he should emphasise that fact, because a certain movement was being started by a few well-meaning people for the establishment of middle-class hospitals, and he was certain that such hospitals could not flourish and exist alone. If provision was ever to be made for that class of people—a class which was worthy of their sympathy, more worthy, perhaps, in some instances than the very poor—it would have to be made otherwise than in hospitals specially constructed and maintained for this purpose. The Hampstead Hospital was started as a pay hospital of that kind, but it was found that the scheme was not successful, and the managers had had the courage to acknowledge this, and to start on a new basis. The result had been that the popularity of the hospital had increased, they received more money from every source, they treated more patients, and, in addition, they were still able to treat in their wards a considerable number of the patients of the class he had been alluding to—about 25 per cent. of the whole number of patients. That hospital had a great claim upon the inhabitants of Hampstead, because it contained a complete idea of what a modern hospital ought to be, seeing that it provided for all classes who required hospital care, except that one class who were rich enough to provide adequate treatment for themselves when ill. (Applause.) Two suggestions he would like to make. First, he thought that the hospital would be better if they did away with the out-patient department and confined their work to in-patients alone. Secondly, the secretary told him that there was a difficulty in getting nurses. That difficulty would disappear if it was made quite plain to nurses that the hospital was able to offer them something that the ordinary hospitals of London could not give them, and that was a knowledge of how to nurse private cases. There should be no difficulty in raising the £10,000 necessary to rebuild the hospital. North London had raised £100,000 to build the Great Northern Central Hospital, and, therefore, he was sure Hampstead could easily raise £10,000. (Applause.)

Mr. E. K. BLYTH responded.

Mr. E. BOND, M.P., proposed the toast of "The Medical Staff," to which Dr. W. HEATH STRANGE responded.

The toast of "The Chairman" concluded the proceedings.

### LONDON TEMPERANCE HOSPITAL.

The twenty-fifth annual report of this institution, which was adopted at a meeting of governors held on Thursday afternoon, April 28th, under the presidency of Mr. CASH, states that during 1897 the number of in-patients received was 1,175, being 18 more than in 1896, and 109 in excess of the total for 1895. Of these in-patients 682 (58 per cent.) were cured, 323 were relieved, 62 unrelieved, and 108 died. The higher death-rate (9·19) is attributed to the extremely large proportion of moribund and semi-moribund cases. "Yet," says the report, "the mortality was lower than the average in London general hospital practice." From the opening of the hospital to December 31st, 1897, last—a period of 24 years and three months—the in-patients attending were 13,984, 8,101 of whom were cured, while 962 died, giving the low death-rate of 6.88 for the whole period. The out-patients in 1897 numbered 6,541, and the visits 18,396, while the casualties were 11,846 and the attendances 24,634. Notable events of the year were the opening of the aseptic ward and of the nurses' home. The inception of the former scheme was due to the late Mr. Frank Wright, and the hospital has to thank Miss Wilson, one of the sisters, for defraying the cost of furnishing the ward. In order to provide additional accommodation for the nurses, Mr. George Cadbury defrayed the cost of purchasing and fitting-up a house in Cardington Street, and also the expense of a new and beautiful dining-room connected with both the hospital and the house. During 1897 alcohol was administered to five patients, and particulars of these exceptional cases are given in the report in accordance with the rules of the institution. Coming to the hospital's finances, the total income of the year, inclusive of special donations of £7,002 to the £10,000 fund, amounted to £15,217. The ordinary expenditure was £9,064, and the extraordinary expenditure £2,133. The sum of £3,961 was transferred to the endowment fund, which now stands at £13,738. The committee refer with satisfaction to the receipt of £647 from the Prince of Wales's Fund, and remark that there is no reason to doubt that the institution will participate in future grants to all general hospitals. On Thursday evening a well-attended public meeting was held at the hospital under the presidency of Mr. Arnold F. Hills, the speakers including Sir Wilfrid Lawson, M.P., Mr. T. W. Russell, M.P., Dr. Collins, L.C.C., and Dr. J. J. Ridge. The last-named gentleman stated in the course of his speech that after twenty-five years' experience they had got to understand that their system was an advantage to the sick. The amount of money spent on intoxicating liquor in the hospitals generally was now very much smaller than when this hospital started. To his mind, this showed that the expenditure on alcohol, thought justifiable twenty-five years ago, was not actually so. Dr. Collins, in referring to the same subject, remarked that their position was strengthened by the existence of the few exceptional cases referred to in the report, because they showed that the staff had an absolutely free hand to administer alcohol where they considered it necessary. In the course of his speech, Mr. T. W. Russell referred to his experience at the Local Government Board and said that of late he had a great deal to do with seeing that pauper nursing should cease in the workhouse infirmaries, and that proper trained nurses should be provided for the unhappy inmates. With regard to the Temperance Hospital, facts must win in the end, and one institution like this in actual operation would influence other hospitals, and the medical profession generally, and so bring about the desired victory. A resolution was passed declaring that the report for 1897 should greatly encourage the friends of the hospital to renewed exertions on its behalf.

### THE HOSPITAL FOR SICK CHILDREN.

In aid of the special appeal for £30,000 which the Hospital for Sick Children is making to purchase the adjoining hospital of St. John and St. Elizabeth, a festival dinner was held at the Whitehall Rooms on Thursday, 28th ult. The Bishop of London presided, and amongst those present were the Duke of Fife (president of the hospital), Lord Aberdare, Earl de la Warr, the Hon. Evelyn Hubbard, M.P., Sir John Wolfe Barry, K.C.B., Sir Henry Burdett, K.C.B, the Hon. Mr. Justice Kekewich, Lieut.-General Sir Andrew Clarke, G.C.M.G., the Archdeacon of London, Mr. Arthur Lucas (chairman of the committee), Mr. John Murray (vice-chairman of the committee), Mr. Guy Pym, M.P., Dr. Thomas Barlow, Mr. Timothy Holmes, the Rev. Dacre Craven, and the Rev. Canon Wace.

The Chairman, in proposing the toast of the evening, "Prosperity to the Hospital for Sick Children," made a most eloquent appeal, in the course of which he said that pain was the great leveller—it made all equal, and therefore, so far as anything could be done to bring it about, the charitable, by means of the hospitals, strove to place at the disposal of the humblest and the poorest all those means of amelioration, and all those resources of science which wealth and plenty could command. If an endeavour was made to do this for the other classes of the community, surely there was no class for whom one would wish to render that service more than for children, to whom pain and all its mystery came with overwhelming force. (Applause.) The Hospital for Sick Children in Great Ormond Street had an exceptional claim upon everyone. It was certainly the largest children's hospital in this country; it was a national institution, and its services were sought from far and wide. Still, though the work it had done was known to everyone and appreciated on all sides, and though it had the very greatest possible claim on everybody's support, and on everybody's sympathy and goodwill—(hear, hear)—it laboured under that difficulty which everything which was quite excellent laboured under—the difficulty that everybody supposed it was everybody else's business to look after it. (Applause.) At the present time there was need that a very exceptional effort should be made to place it upon a firm foundation for the future. It had been found necessary to acquire the adjoining site, and it was to help to raise the £30,000 required for this purpose that he was appealing that night. (Applause.)

The Duke of Fife, in responding, said that if they had not acted promptly and acquired the site of the adjoining hospital of St. John and St. Elizabeth, that site would have been utilised in such a manner as to have affected the very existence of the hospital.

Sir Henry Burdett proposed "The Committee of Management," and said that he noticed with great pleasure that one of the vice-presidents of the hospital was H.R.H. the Princess of Wales, because it was certainly a remarkable sight to see her influence over children, and to realise how fully her affection for them was instinctively returned by the little ones. Her Royal Highness had felt that this should be made use of in order to help the hospitals of London; she had felt that since the Prince of Wales started his Hospital Fund there had been too much of the gentleman in it, and that the ladies ought to take some part in it as well. Accordingly she had, on her own initiative, written a letter to the children of England impressing upon them the privilege of giving their pennies to support the hospitals through the Prince of Wales's Fund. He mentioned this fact because it was useful sometimes when meeting people interested in an individual hospital to show them that they had a responsibility towards a great movement which aimed at helping the whole of the hospitals of the metropolis. The president, the Duke of Fife, had very kindly allowed his children to be photographed for the Roll of Ministering Children, and if those who were interested in The Hospital for Sick Children would allow their children to be photographed for that Roll they would benefit not only the hospitals of London but the finances of The Hospital for Sick Children. He also pointed out that the committee seemed to have overlooked the fact that the grant by the Prince of Wales's Fund to the hospital this year contained what was practically an annual subscription of £500 a year. (Applause.) After instancing various kinds of committees of management with which he had come in contact during the many years he had been engaged in hospital work, he said that the success of The Hospital for Sick Children and the general efficiency of the institution (to which he could speak with knowledge) was due in great measure to the fact that some of the most representative and best known men in London were on that committee, and that they had discharged their duties adequately and well. (Applause.)

Mr. Arthur Lucas (chairman of the committee), in responding, detailed the reasons which had rendered it imperative that the adjoining site should be acquired, and the steps which had been taken so far to raise the money to defray the cost. Referring to the out-patient question, he said that he believed that if the Metropolitan provident dispensaries could only be made to work in touch with the hospitals of London, a great step in the direction of solving the problem of the out-patient department would have been made. The medical men who worked for the provident dispensaries were able men, but, as a rule, they were men whose names were not known, whilst the medical men who worked on the staffs of the hospitals were men who were well-known and had been probably the most brilliant men in the schools. In those circumstances it was not human nature for a man to pay 6d. to the unknown man at the provident dispensary when he could get for nothing the highest advice in the out-patient department of the voluntary hospital.

The Secretary announced that the total collection amounted to £7,578, made up as follows: Towards the purchase of the Hospital of St. John and St. Elizabeth £4,500; for the endowment of cots, £3,000; in new annual subscriptions, £70; donations for hospital maintenance, £8.

Sir J. WOLFE BARRY proposed, and Dr. THOMAS BARLOW, the senior physician, responded to the toast of "The Medical Officers."

The proceedings shortly afterwards terminated.

### LONDON LOCK HOSPITAL.

Lord KINNAIRD presided at the 151st annual meeting of the London Lock Hospital and Rescue Home, held at Dean Street, Soho, on Thursday, 28th ult. Referring to the falling off in the income for the year of £820, as compared with 1896, his Lordship remarked that they had suffered in common, he supposed, with most charities, owing to the Jubilee; people appeared to have spent so much on themselves that they had less to spare for such institutions. The accounts of the three branches—Female Hospital, Rescue Home and Male Hospital, and Out-patients' Department—showed total receipts for the year of £7,606, as against a total expenditure of £8,836, and a deficiency of £1,302, as compared with a balance on the wrong side of only £66 in 1896. This was largely due to exceptionally heavy expenditure in painting, and in additional laundry machinery. From the Prince of Wales's Fund they had received £122, from the Hospital Sunday Fund £122, and from the Hospital Saturday Fund £119. The trustees of the Marriott Fund had made them a grant of £1,000, and though thankful for this they had hoped it would have been £2,000. Indeed, they still hoped the trustees would see their way to double the grant. With regard to the work of the hospital, the report shows that 694 in-patients were admitted to the female hospital, and 283 to the male hospital; while the out-patients numbered 22,233, 20,257 being males, and 1,976 females. Of the female in-patients, 72 passed into the home, a good many more were admitted into other homes, 49 had been placed in respectable situations, and six restored to their friends. Special reference was made to the kindness of the Rev. Charles Chapman, who having opened the St. Mildred's Convalescent Home at Bexhill-on-Sea, has generously placed a bed at the disposal of the governors. The ordinary convalescent home will not admit these cases, and the governors have no funds to open a special home themselves. The only expense in cases sent to Bexhill is the travelling to and fro, which is taken from the Samaritan Fund. The adoption of the report, and the usual formal resolutions were duly carried.

### KING'S COLLEGE HOSPITAL.

King's College Hospital festival dinner was held at the Whitehall Rooms, on Tuesday, the 3rd inst. Lord LISTER, P.R.S., presided, and was supported by, amongst others, Viscount Dillon (chairman of the committee), Sir John Bridge, Sir Elliott Lees, Bart., M.P., Sir Archibald Giekie, Mr. A. H. A. Morton, M.P., the Rev. Dr. Robertson (vice-chairman of the committee), Mr. Charles Awdrey (treasurer), and the Rev. Dr. Wace. The Chairman, after proposing the usual loyal toasts, gave the toast of "Success to the Hospital," and in doing so said that he would be very ungrateful if he did not sincerely feel the wish that this toast expressed. It was very many years since King's College had done him the great honour of inviting him to be a member of the staff of the institution, and during the six years that he served on that staff he experienced uniform kindness and consideration. (Hear, hear.) He was pleased to know that never had King's College Hospital more discharged its great function of relieving the physical ills of humanity than it did at the present time. The hospital at the present moment had several special wants. The flooring of the wards required renewal, a circumstance which would entail a very large expenditure in the near future. Then, too, although electric light had been introduced with great advantage into the operating theatre and the ophthalmic department, it was very desirable that it should be introduced throughout the building, and for this £1,000 at least would be required. The out-patient department also was not in such an excellent condition as it should be, and finally the convalescent home was £1,000 in debt. Altogether, therefore, he had good grounds for begging those present to make on this occasion very liberal contributions to the hospital. (Applause. Referring to the nursing arrangements, Lord Lister said that there was no hospital in London which was superior in this matter to King's College Hospital, thanks to the admirable administration of the excellent matron. (Loud applause. Viscount Dillon responded. Mr. A. H. A. Morton, M.P., proposed "The National Services," a toast to which the Rev. Dr. Wace, Mr. G. W. Bell, and Sir Elliott Lees, Bart., responded. Other toasts followed. The contributions amounted to £3,025, including £1,000 given by Mr. Ravenscroft for the endowment of a bed, £300 from Messrs. W. H. Smith and Sons, and £100 from Lord Lister.

